# Associations between plasma metal mixture exposure and risk of hypertension: A cross-sectional study among adults in Shenzhen, China

**DOI:** 10.3389/fpubh.2022.1039514

**Published:** 2022-12-13

**Authors:** Sijia Zheng, Zhiqiang Nie, Ziquan Lv, Tian Wang, Weizhou Wei, Daokui Fang, Xuan Zou, Yulin Fu, Tingting Cao, Zhaoyi Liang, Qi Lu, Hui Huang, Ying Wen, Suli Huang

**Affiliations:** ^1^School of Public Health, Shanxi Medical University, Taiyuan, China; ^2^Shenzhen Center for Disease Control and Prevention, Shenzhen, China; ^3^Department of Cardiology, Guangdong Cardiovascular Institute, Guangdong Provincial People's Hospital, Guangdong Academy of Medical Sciences, Guangzhou, China; ^4^Key Laboratory of Environmental Medicine Engineering, Ministry of Education, School of Public Health, Southeast University, Nanjing, China; ^5^Shenzhen Yutian Community Health Service Centre, Shenzhen, China; ^6^Department of Cardiology, The Eighth Affiliated Hospital, Sun Yat-sen University, Shenzhen, China

**Keywords:** hypertension, metal mixture, cobalt, aluminum, calcium, BKMR

## Abstract

**Background:**

Metal exposure affects human health. Current studies mainly focus on the individual health effect of metal exposure on hypertension (HTN), and the results remain controversial. Moreover, the studies assessing overall effect of metal mixtures on hypertension risk are limited.

**Methods:**

A cross-sectional study was conducted by recruiting 1,546 Chinese adults who attended routine medical check-ups at the Eighth Affiliated Hospital of Sun Yat-sen University, Shenzhen. The plasma levels of 13 metals were measured using inductively coupled plasma mass spectrometry. Multivariate logistic regression model, restricted cubic spline (RCS) model and the Bayesian Kernel Machine Regression (BKMR) model were applied to explore the single and combined effect of metals on the risk of HTN.

**Results:**

A total of 642 (41.5%) participants were diagnosed with HTN. In the logistic regression model, the adjusted odds ratios (ORs) were 0.71 (0.52, 0.97) for cobalt, 1.40 (1.04, 1.89) for calcium, 0.66 (0.48, 0.90), and 0.60 (0.43, 0.83) for aluminum in the second and third quartile, respectively. The RCS analysis showed a V-shaped or an inverse V-shaped dose-response relationship between metals (aluminum or calcium, respectively) and the risk of HTN (P for non-linearity was 0.017 or 0.009, respectively). However, no combined effect was found between metal mixture and the risk of hypertension.

**Conclusions:**

Plasma levels of cobalt, aluminum and calcium were found to be associated with the risk of HTN. Further studies are needed to confirm our findings and their potential mechanisms with prospective studies and experimental study designs.

## 1. Introduction

Hypertension (HTN) is one of the major risk factors for many severe diseases (1). It is estimated that there are more than 1 billion people with HTN worldwide since 2019, doubling the number of 1990 (1). According to data from the Million People Program (MPP), the prevalence of HTN in adults of China aged from 35 to 75 years is about 44.7%, among which only 7.2% had achieved control with prescribed medications (2). Globally, high systolic blood pressure was the major secondary risk factor for attributable deaths in 2019, accounting for 10.8 million deaths (3). Therefore, HTN continues to be a major global public health burden, and the prevention and control remain critical. Currently, genetics, dietary and lifestyles are well-established risk factors in relation to the etiology of HTN (1), while amounting studies have indicated that the metal exposure might also be responsible for the occurrence of HTN (4).

Humans can be exposed to metals through various routes (e.g., air inhalation, food digestion and skin contact) (5). The essential elements might play an indispensable role in human physiological activities, including immunity, metabolism and development (6). Nevertheless, excess or deficient amounts of essential elements might also exert adverse effects on human health (6, 7). The toxic metals can disrupt body homeostasis and cause organ damage (8). Amounting epidemiological investigations have focused on the effects of metal exposure on HTN, but the results are still inconclusive. The positive correlation was found between cadmium levels in the blood and HTN prevalence in Korean adults (8). However, a cross sectional study conducted in Canada (4) showed that urinary cadmium was inversely correlated with blood pressure among the general adults. Zhong et al. (9) indicated that the urinal level of zinc was positively associated with the prevalence of HTN in adults of China, while a cross-sectional study of adults in Pakistan (7) found that zinc deficiency might have a synergistic effect with risk factors related to HTN. The conflicting findings between studies could be due to different internal exposure biomarker, exposure levels or study designs, therefore warranting further studies to confirm the results.

Humans are often exposed to metals in the form of mixtures that may be correlated with each other and jointly affect human health (10). However, most researches have only focused on the individual health effect of metal exposure on HTN (4, 8), and the studies using multi-pollutant statistical methods [such as Bayesian kernel machine regression (BKMR) and weighted quantile sum (WQS)] to evaluate the overall effects of metal mixture exposure on health are limited, especially in Chinese populations (10). Only one study in China (9) investigated the association between urinary polymetallic mixtures and the risk of HTN in adults along the Yangtze River. BKMR analysis revealed a significant association effect of five metals (Cd, Cu, Mg, Mo, and Zn) on HTN. BKMR analysis is a more advanced statistical method for analyzing the combined effects or potential interactions between multiple environmental pollutants and disease risk in epidemiological studies (11). However, the evidence in Southern China is missing. Given the high burden of metal exposure and HTN prevalence in the populations in southern China (12, 13), we applied diverse statistical methods in this study to explore the individual and combined effect of plasma metals on risk of HTN in the general adult population in Southern China.

## 2. Materials and methods

### 2.1. Study participants

In this study, all the participants were adults recruited from people who underwent routine medical check-ups organized by their employers in the Eighth Affiliated Hospital of Sun Yat-sen University in Shenzhen between October 2012 and April 2017. We excluded the participants that had neurological disorder, malignancy, cerebrovascular disease or peripheral arterial occlusive disease. Since we supposed that people above 30 years old might be more prone to HTN or other chronic diseases, so a total of 1,720 subjects above 30 years old were detected metal levels. For statistical analysis, the participants with missing data on blood pressure (*n* = 135), age (*n* = 1), glomerular filtration rate (eGFR) (*n* = 10), uric acid (UA) (*n* = 27), and diabetes (*n* = 1) were further excluded. Finally, a total of 1,546 adults aged 31–91 years were included in this study, including 642 individuals with HTN (Supplementary Figure S1). Our study was approved by the Ethics Committee of the Shenzhen Center for Disease Control and Prevention. The informed consent forms were collected from each participant at the survey.

### 2.2. Data collection

Data were collected with structured questionnaires. Trained interviewers collected general demographic characteristics and potential risk factors, including gender, age, height, weight, lifestyle habits, and disease history, through face-to-face interviews. The definitions of smokers, drinkers, hyperlipidaemia and diabetes were described in detail in previous studies, with specific information in Supplementary Appendix S1. Fasting blood samples were collected from participants using vacuum blood collection tubes (BD, USA). Blood glucose, lipid and uric acid levels were measured using the biochemical automatic detector (Beckman AU5800). All biological samples were stored at −80°C prior to analysis.

According to the 2018 Chinese Guidelines for Prevention and Treatment of HTN (14), HTN was defined with a systolic blood pressure ≥140 mmHg or diastolic blood pressure ≥90 mmHg, self-reported physician diagnosis or currently using anti-hypertensive medication. In this study, the blood pressures of participants were measured by a professional nurse. In brief, participants were asked to take two blood pressure measurements after a 5-min break using a regularly calibrated sphygmomanometer. The final blood pressure value was the average of the two blood pressure readings.

The estimated glomerular eGFR was calculated based on the Modification of Diet in Renal Disease (MDRD) study formula for Chinese adults (15, 16). The formula is as follows:

MDRD: eGFR = 175 × SCr^−1.234^ × age^−0.179^ [if female, ×0.79]

In the above equation, the serum creatinine (SCr) is serum creatinine in mg/dL, age is in years.

### 2.3. Measurement of plasma metals

We measured plasma levels of 13 metals, including magnesium (Mg), manganese (Mn), cobalt (Co), aluminum (Al), iron (Fe), calcium (Ca), copper (Cu), zinc (Zn), arsenic (As), selenium (Se), molybdenum (Mo), cadmium (Cd), and thallium (Tl), using inductively coupled plasma mass spectrometry (ICP-MS, Agilent 7700 series, Agilent Technologies, USA). A previous report (17) and Supplementary Appendix S2 describe the details. Briefly, blood samples were transferred to the polypropylene centrifuge tubes and acidified at room temperature with 300 μL of 55% (v/v) HNO3. Following cooling in a boiling water bath, the samples were diluted with ultrapure water and analyzed. We used certified reference materials (ClinChek Human Plasma Control Trace Elements No. 8883 and No. 8884, Recipe, Munich, Germany) to assess the accuracy and precision of the assay. Instrument performance was evaluated every 30 samples during the experiment. If the results were suggestive of potential sample contamination or abnormal measurements, the instrument would be recalibrated and re-analyzed. The intra- and inter-assay coefficients of variation (CVs) for plasma metals were shown in Supplementary Table S1. Specifically, the intra- and inter-assay CVs were 0.05 and 0.08% for Mg, 7.20 and 8.97% for Mn, 4.39 and 5.87% for Co, 4.75 and 6.94% for Al, 5.92 and 7.47% for Fe, 0.06 and 0.08% for Ca, 3.76 and 4.39% for Cu, 3.77 and 4.08% for Zn, 7.55 and 8.71% for As, 3.48 and 4.54% for Se, 1.41 and 6.48% for Mo, 8.61 and 7.17% for Cd, 7.48 and 8.36% for Tl. In addition, the detection rates and limits of detection (LOD) for all plasma metals were also shown in Supplementary Table S1. Measurements below the LOD were noted as the LOD divided by the square root of 2 (17).

### 2.4. Statistical analysis

General demographic characteristics and metal concentrations of participants were presented as frequencies (percentages), means ± SD or medians (IQR). Statistics for differences between the two groups were performed using the Student's *t*-test, the Wilcoxon rank sum test, or a chi-square test depending on the distribution of the data. A natural transformation of all plasma metals was performed to approach a normal distribution. The correlation between metals was examined by Spearman's rank correlation analysis.

Estimates of the odds ratios (ORs) and 95% confidence intervals (CIs) between plasma metal exposure and risk of HTN were calculated using the single-metal logistic regression models. Each plasma metal was grouped into quartiles based on the distribution of the non-hypertensive group. We identified the relevant covariates between metals and HTN by reviewing the literature and using directed acyclic graphs (DAG, http://www.dagitty.net/dags.html). By plotting the DAG (Supplementary Figure S2), covariates in model 1 included sex, age, body mass index (BMI), smoking and drinking, and the covariates including diabetes, hyperlipidemia, eGFR, uric acid (UA) and family history of HTN were additionally adjusted in model 2. The median level of each quartile metal was used as a continuous variable for the trend test. A Benjamini-Hochberg false discovery rate (FDR) correction was used in the regression model to adjust for multiple testing (α = 0.1), which sorted the *p*-values in ascending order and then divided each metal *p*-value by its percentile rank to obtain its corresponding estimated *p*-FDR. Furthermore, we calculated E-values according to ORs of model 2 to assess the potential impact of unmeasured confounders (18). Multiple linear regression analysis was used to analyze the association between metals and blood pressure values.

The metals that were significantly associated with the risk of HTN in the single metal model were included in the multi-metal model and analyzed by stepwise regression, adjusting for the same covariates as model 2. The multicollinearity of the estimated metal was assessed with variance inflation factor (VIF), for which multicollinearity may not affect the estimation when VIF < 10 (19). Moreover, to explore the dose-response relationship between plasma metals and HTN risk, we applied restricted cubic spline (RCS) regression models. Knots was set at the 10th, 50th, and the 90th percentile of metal concentrations among the whole study population, and the reference value was set at the 10th percentile.

The joint effects and interactions of metal mixtures on the risk of HTN were further assessed by the BKMR model (11). Detailed description of the model has been described in one previous study (20). Briefly, the method, based on a high Markov chain Monte Carlo algorithm, was implemented for 25,000 iterations. Furthermore, a univariate effects model was used to assess the association of a single element with HTN by changing each metallic element from its 25th percentile to its 75th percentile, while keeping the other metallic elements at the 25th, 50th, or 75th percentile.

Statistical software SPSS 25.0 (Statistical Package for the Social Science, IBM SPSS Inc., Chicago, IL, USA) and R software (“rcssci” package; version 4.2.2; Lucent Technologies, USA) were used for analysis, and all two-tailed *p* < 0.05 were considered statistically significant.

## 3. Results

### 3.1. Study participant characteristics

The general characteristics of the study participants and the distribution of plasma metal concentrations were summarized in Table 1. Among the participants, about 41.53% (642/1,546) participants showed HTN. Compared with the non-hypertensive group, participants with HTN were older and had higher BMI, triglyceride (TG,) uric acid (UA), SBP, DBP levels and the prevalence of diabetes and hyperlipidemia (*p* < 0.05), while the eGFR level was lower in the hypertensive group (*p* < 0.001). Participants with HTN showed higher plasma levels of Cu, As, and Se (*p* = 0.016, 0.011, and 0.005, respectively), while concentrations of Co and Ca were lower (*p* < 0.001). The proportion of metals that were not detectable (<LOD) was <6.5% in all samples (Supplementary Table S1). The correlation between plasma metals were shown in Supplementary Figure S3. The range of Spearman rank correlation coefficient was −0.22 to 0.52.

**Table 1:** General characteristics and plasma metal concentrations of participants in the study.

**Variable**	**Total population (*n* = 1,546)**	**Non-hypertension (*n* = 904)**	**Hypertension** **(*n* = 642)**	***P-*value**
Age, years	59.23 ± 9.55	55.88 ± 9.60	62.52 ± 8.45	< 0.001
Male, *n* (%)	701 (45.3)	420 (46.5)	281 (43.8)	0.320
BMI, kg/m^2^	23.81 ± 2.61	23.51 ± 2.74	24.25 ± 2.84	< 0.001
Smoking, *n* (%)	194 (12.5)	136 (15.0)	58 (9.0)	< 0.001
Alcohol drinking, *n* (%)	180 (11.6)	106 (11.7)	74 (11.5)	0.964
TC, mmol/L	5.39 (4.65, 6.08)	5.39 (4.65, 6.03)	5.38 (4.66, 6.15)	0.804
TG, mmol/L	1.27 (0.89, 1.82)	1.19 (0.84, 1.74)	1.38 (0.98, 1.98)	0.005
HDL-c, mmol/L	1.37 (1.15, 1.61)	1.38 (1.15, 1.62)	1.36 (1.10, 1.60)	0.431
LDL-c, mmol/L	3.08 (2.52, 3.71)	3.08 (2.52, 3.68)	3.09 (2.52, 3.77)	0.786
eGFR, ml/min	99.27 (85.10, 114.47)	101.06 (87.57, 116.46)	96.80 (82.58, 111.89)	< 0.001
UA, μmol/L	331.00 (282.00, 392.00)	321.00 (273.25, 375.00)	351.00 (294.00, 410.00)	< 0.001
Diabetes, *n* (%)	140 (9.1)	51 (5.6)	89 (13.9)	< 0.001
Hyperlipidemia, *n* (%)	166 (10.7)	68 (7.5)	98 (15.3)	< 0.001
Family history of hypertension, *n* (%)	558 (36.1)	308 (34.1)	250 (38.9)	0.106
SBP, mmHg	129.00 (119.00, 139.00)	124.00 (116.00, 132.00)	139.00 (127.00, 150.00)	< 0.001
DBP, mmHg	81.00 (74.00, 88.00)	79.00 (73.00, 84.00)	86.00 (77.00, 93.00)	< 0.001
**Metal concentration**, **μg/L**
Magnesium	20,570.65 (18,823.48, 22,325.68)	20,529.23 (18,772.86, 22,259.79)	20,612.85 (18,983.41, 22,411.22)	0.411
Manganese	0.94 (0.60, 1.44)	0.92 (0.61, 1.42)	0.95 (0.59, 1.47)	0.705
Cobalt	0.24 (0.20, 0.29)	0.25 (0.21, 0.30)	0.24 (0.18, 0.29)	< 0.001
Aluminum	23.90 (16.97, 37.29)	24.59 (18.10, 36.70)	23.02 (15.95, 37.93)	0.062
Iron	1,693.43 (1,313.75, 2,204.25)	1,715.35 (1,313.58, 2,235.11)	1,659.43 (1,314.59, 2,167.95)	0.529
Calcium	79,798.37 (75,143.44, 86,986.65)	80,341.21 (75,482.88, 89,072.32)	79,268.16 (74,724.47, 84,129.33)	< 0.001
Copper	919.65 (777.76, 1,052.70)	916.97 (759.62, 1,047.69)	929.97 (809.60, 1,056.94)	0.016
Zinc	1,049.01 (884.31, 1,238.52)	1,031.16 (860.71, 1,240.65)	1,067.39 (909.27, 1,235.26)	0.063
Arsenic	1.14 (0.64, 2.08)	1.07 (0.62, 1.90)	1.25 (0.70, 2.26)	0.011
Selenium	98.93 (87.14, 113.66)	97.06 (85.72, 112.37)	100.69 (89.44, 115.31)	0.005
Molybdenum	1.07 (0.87, 1.34)	1.06 (0.85, 1.31)	1.09 (0.89, 1.37)	0.083
Cadmium	0.05 (0.03, 0.07)	0.05 (0.03, 0.07)	0.05 (0.03, 0.07)	0.339
Thallium	0.11 (0.09, 0.15)	0.11 (0.09, 0.15)	0.11 (0.09, 0.14)	0.424

BMI, body mass index; TC, total cholesterol; TG, triglyceride; HDL-c, high-density lipoprotein cholesterol; LDL-c, low-density lipoprotein cholesterol; eGFR, nephron glomerular filtration rate; UA, uric acid; SBP, systolic blood pressure; DBP, diastolic blood pressure. Data are presented as number (percentage) for categorical data, mean ± standard deviation for parametrically distributed data or median (interquartile range) for non-parametrically distributed data. Students's *t*-test or Wilcoxon rank sum test were performed for comparison of the continuous variables according to the data distribution, and Chi-square test for the categorical variables.

### 3.2. Association between plasma metal exposure and HTN/blood pressure

The association between plasma metals and the risk of HTN by logistic regression models were shown in Table 2. Compared with the first quartile (model 1), the ORs (95%CIs) were 0.72 (0.53, 0.98) for Co, 1.53 (1.10, 2.13) for Cu, and 0.73 (0.53, 0.99) for Cd in the second quartile, and ORs (95% CIs) were 0.61 (0.44, 0.83) for Al and 1.41 (1.03, 1.94) for Zn in the third quartile. After additional adjustment for diabetes, hyperlipidemia, family history of HTN, eGFR and UA (model 2), the adjusted ORs (95% CI) was 0.71 (0.52, 0.97) for Co and 1.40 (1.04, 1.89) for Ca comparing the second with the first quartile of metals. Compared with the first quartile, participants in the second and third quartiles of Al concentration had a 34% (95% CI: 0.48, 0.90) and 40% (95% CI: 0.43, 0.83) reduction in the risk of HTN, respectively. The E-values of ORs of model 2 were 1.66 (Co), 1.91 (Al), and 1.65 (Ca), respectively (Supplementary Figure S4), indicating that the effect of unmeasured confounders would have to be larger than these elements to explain the association with HTN risk, confirming that the association between the three metals and HTN risk remained robust in the absence of the non-measured confounders.

**Table 2:** Odds ratios (95%CI) for the risk of hypertension associated with quartiles of plasma metals based on the single-metal model.

**Metal**	**Metal concentrations (**μ**g/L)**^**a**^				***P*-value**	***p*-FDR^b^**
	**Q1**	**Q2**	**Q3**	**Q4**		
Single-metal model						
Mg	≤ 18,772.86	18,772.86–20,529.23	20,529.23–22,259.79	>22,259.79		
*n* (controls/cases)	226/145	226/166	226/158	226/173		
Crude	1.00 (Ref)	1.13 (0.85, 1.51)	1.08 (0.80, 1.44)	1.19 (0.89, 1.59)	0.661	0.661
Model 1	1.00 (Ref)	0.99 (0.72, 1.35)	0.89 (0.65, 1.22)	0.93 (0.68, 1.27)	0.890	0.890
Model 2	1.00 (Ref)	0.90 (0.65, 1.24)	0.82 (0.59, 1.14)	0.89 (0.65, 1.24)	0.763	0.763
Mn	≤ 0.61	0.61–0.92	0.92–1.42	>1.42		
*n* (controls/cases)	226/166	226/139	226/166	226/171		
Crude	1.00 (Ref)	0.84 (0.63, 1.12)	1.00 (0.75, 1.33)	1.02 (0.77, 1.36)	0.496	0.586
Model 1	1.00 (Ref)	1.05 (0.77, 1.44)	1.24 (0.91, 1.68)	1.05 (0.78, 1.42)	0.568	0.672
Model 2	1.00 (Ref)	1.10 (0.80, 1.51)	1.36 (0.99, 1.88)	1.17 (0.85, 1.61)	0.287	0.414
Co	≤ 0.21	0.21–0.25	0.25–0.30	>0.30		
*n* (controls/cases)	217/220	217/132	218/146	217/137		
Crude	1.00 (Ref)	0.60 (0.45, 0.80)	0.66 (0.50, 0.87)	0.63 (0.48, 0.84)	0.001	0.002
Model 1	1.00 (Ref)	0.72 (0.53, 0.98)	0.79 (0.59, 1.07)	0.81 (0.60, 1.10)	0.117	0.216
Model 2	1.00 (Ref)	0.71 (0.52, 0.97)	0.83 (0.61, 1.13)	0.86 (0.63, 1.18)	0.172	0.334
Al	≤ 18.10	18.10–24.59	24.59–36.70	>36.70		
*n* (controls/cases)	220/213	221/127	221/115	220/169		
Crude	1.00 (Ref)	0.60 (0.45, 0.80)	0.54 (0.40, 0.72)	0.79 (0.60, 1.04)	< 0.001	0.001
Model 1	1.00 (Ref)	0.71 (0.52, 0.96)	0.61 (0.44, 0.83)	0.84 (0.63, 1.13)	0.012	0.076
Model 2	1.00 (Ref)	0.66 (0.48, 0.90)	0.60 (0.43, 0.83)	0.82 (0.61, 1.11)	0.006	0.067
Fe	≤ 1,313.58	1,313.58–1,715.35	1,715.35–2,235.11	>2,235.11		
*n* (controls/cases)	223/158	223/181	223/158	223/137		
Crude	1.00 (Ref)	1.15 (0.86, 1.52)	1.00 (0.75, 1.33)	0.87 (0.65, 1.16)	0.312	0.406
Model 1	1.00 (Ref)	1.20 (0.89, 1.64)	1.07 (0.78, 1.47)	0.90 (0.65, 1.26)	0.318	0.459
Model 2	1.00 (Ref)	1.24 (0.91, 1.70)	1.08 (0.78, 1.50)	0.96 (0.68, 1.35)	0.383	0.498
Ca	≤ 75,482.88	75,482.88–80,341.21	80,341.21–89,072.32	>89,072.32		
*n* (controls/cases)	226/184	226/194	226/160	226/104		
Crude	1.00 (Ref)	1.05 (0.80, 1.39)	0.87 (0.66, 1.15)	0.57 (0.42, 0.77)	< 0.001	0.002
Model 1	1.00 (Ref)	1.32 (0.98, 1.77)	1.11 (0.82, 1.50)	0.74 (0.53, 1.02)	0.005	0.064
Model 2	1.00 (Ref)	1.40 (1.04, 1.89)	1.21 (0.88, 1.65)	0.82 (0.58, 1.16)	0.010	0.067
Cu	≤ 759.62	759.62–916.97	916.97–1,047.69	>1,047.69		
*n* (controls/cases)	225/107	225/199	225/162	225/171		
Crude	1.00 (Ref)	1.88 (1.39, 2.53)	1.53 (1.12, 2.08)	1.60 (1.18, 2.18)	0.001	0.002
Model 1	1.00 (Ref)	1.53 (1.10, 2.13)	1.10 (0.78, 1.56)	1.18 (0.83, 1.68)	0.052	0.168
Model 2	1.00 (Ref)	1.40 (0.99, 1.96)	1.08 (0.76, 1.54)	1.13 (0.79, 1.62)	0.205	0.334
Zn	≤ 860.71	860.71–1,031.16	1,031.16–1,240.65	>1,240.65		
*n* (controls/cases)	226/115	226/164	226/206	225/155		
Crude	1.00 (Ref)	1.41 (1.04, 1.90)	1.78 (1.33, 2.39)	1.34 (0.99, 1.81)	0.002	0.004
Model 1	1.00 (Ref)	1.16 (0.84, 1.60)	1.41 (1.03, 1.94)	1.00 (0.72, 1.39)	0.079	0.177
Model 2	1.00 (Ref)	1.10 (0.79, 1.54)	1.29 (0.93, 1.80)	0.91 (0.65, 1.28)	0.140	0.334
As	≤ 0.62	0.62–1.07	1.07–1.90	>1.90		
*n* (controls/cases)	225/127	225/136	225/192	225/185		
Crude	1.00 (Ref)	1.05 (0.77, 1.43)	1.29 (0.96, 1.74)	1.45 (1.08, 1.95)	0.006	0.011
Model 1	1.00 (Ref)	0.97 (0.70, 1.35)	1.19 (0.86, 1.64)	1.30 (0.95, 1.78)	0.210	0.341
Model 2	1.00 (Ref)	1.00 (0.71, 1.41)	1.16 (0.83, 1.61)	1.23 (0.89, 1.70)	0.519	0.613
Se	≤ 85.72	85.72–97.06	97.06–112.37	>112.37		
*n* (controls/cases)	224/135	225/159	225/159	224/180		
Crude	1.00 (Ref)	1.05 (0.78, 1.43)	1.50 (1.12, 2.00)	1.44 (1.07, 1.92)	0.003	0.006
Model 1	1.00 (Ref)	0.89 (0.64, 1.24)	1.36 (0.99, 1.85)	1.23 (0.90, 1.69)	0.033	0.142
Model 2	1.00 (Ref)	0.86 (0.61, 1.20)	1.27 (0.93, 1.76)	1.16 (0.84, 1.62)	0.072	0.312
Mo	≤ 0.85	0.85–1.06	1.06–1.31	>1.31		
*n* (controls/cases)	224/135	225/159	225/159	224/180		
Crude	1.00 (Ref)	1.19 (0.89, 1.60)	1.19 (0.89, 1.60)	1.34 (1.01, 1.80)	0.061	0.099
Model 1	1.00 (Ref)	1.04 (0.76, 1.43)	0.94 (0.68, 1.29)	0.91 (0.66, 1.26)	0.849	0.890
Model 2	1.00 (Ref)	1.06 (0.77, 1.46)	0.91 (0.65, 1.27)	0.88 (0.63, 1.23)	0.668	0.724
Cd	≤ 0.03	0.03–0.05	0.05–0.07	>0.07		
*n* (controls/cases)	218/178	219/130	219/177	218/146		
Crude	1.00 (Ref)	0.73 (0.55, 0.98)	1.00 (0.75, 1.32)	0.82 (0.61, 1.09)	0.096	0.138
Model 1	1.00 (Ref)	0.73 (0.53, 0.99)	1.06 (0.78, 1.43)	0.98 (0.72, 1.34)	0.082	0.177
Model 2	1.00 (Ref)	0.78 (0.56, 1.07)	1.14 (0.83, 1.55)	1.03 (0.75, 1.43)	0.115	0.334
Tl	≤ 0.09	0.09–0.11	0.11–0.15	>0.15		
*n* (controls/cases)	225/178	226/147	226/155	225/154		
Crude	1.00 (Ref)	0.83 (0.62, 1.10)	0.87 (0.66, 1.16)	0.87 (0.65, 1.15)	0.563	0.610
Model 1	1.00 (Ref)	0.81 (0.60, 1.11)	0.83 (0.61, 1.13)	0.89 (0.66, 1.21)	0.487	0.634
Model 2	1.00 (Ref)	0.74 (0.54, 1.02)	0.75 (0.55, 1.03)	0.81 (0.59, 1.11)	0.187	0.334

a Plasma metal concentrations are shown as raw data.

Model 1 was obtained from logistic regression models with adjustment for age, sex, BMI, smoking, and drinking.

Model 2 was additionally adjusted for UA, family history of hypertension, diabetes, hyperlipidemia, and eGFR.

b Values were obtained from logistic regression models when median in each quartile of log-transformed metal level entered models as continuous variable. FDR corrections were performed to adjust for multiple tests.

The correlation between plasma metal and blood pressure was further analyzed by multiple linear regression models (Supplementary Table S2). After adjustment for confounders, Al was negatively correlated with SBP (β = −1.565, 95% CI: −3.000, −0.129; *p* = 0.033) and DBP (β = −1.294, 95% CI: −2.210, −0.378; *p* = 0.006).

In the multi-metal model that included all three metals (Co, Al, and Ca) simultaneously, the results (Supplementary Figure S5) were consistent with those of the single-metal model. Compared to the lowest quartile, the ORs for Co and Ca in the second quartile were 0.69 (95% CI: 0.50–0.96) and 1.40 (95% CI: 1.04–1.89), respectively, while the ORs for Al in the second and third quartiles were 0.71 (95% CI: 0.52–0.97) and 0.66 (95% CI: 0.48–0.90). The VIFs in the model were all <1.1.

### 3.3. Plasma metals and risk of HTN dose-response relationships

**Figure 1:**
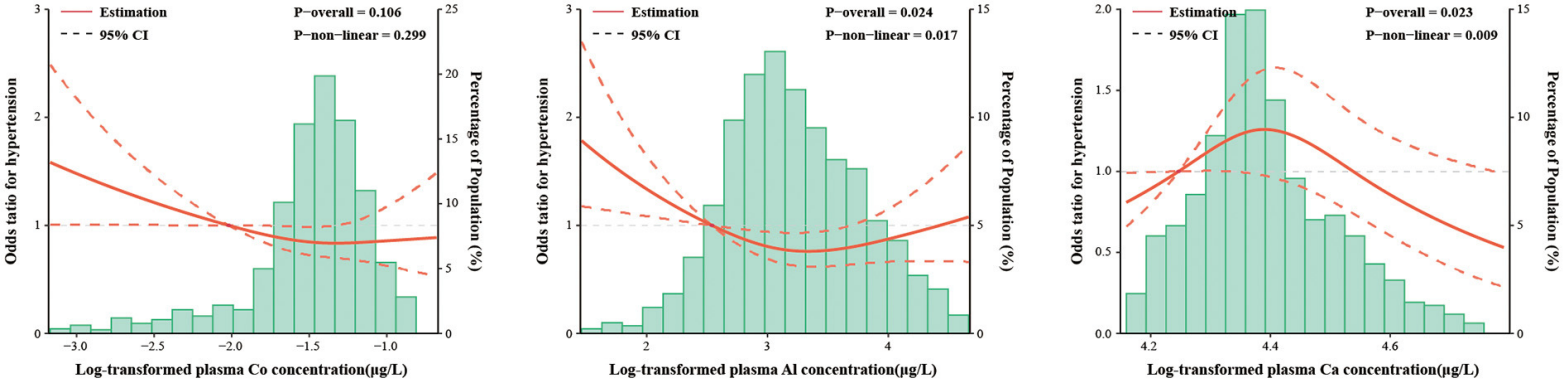
The restricted cubic spline for the associations between plasma metal concentrations and the risk of hypertension. Co, Cobalt; Al, Aluminum; Ca, Calcium. The lines represent adjusted odds ratios (solid lines) and 95% confidence intervals (long dashed lines) based on the restricted cubic spline models for the ln-transformed concentrations of plasma cobalt, aluminum and calcium. The reference values were set at 10th percentiles, and the knots were set at 10th, 50th, and 90th percentiles of the ln-transformed concentrations, respectively. Adjusted factors were consistent with model 2. Plasma metal concentrations with ln-transformation are presented on the *X*-axis. The histograms represent the distributions of plasma metal concentrations among the study population, excluding the values outside the 5th and 95th percentiles.

To further explore the dose-response relationships between metals and the HTN risk, the RCS model was applied based on the results of the logistic regression models, which included Co, Ca and Al. As shown by the curves in Figure 1, plasma levels of Al were associated with the risk of HTN in a V-shaped dose-response relationship (*p* for overall association = 0.024, non-linearity *p* = 0.017), while plasma levels of Ca were correlated with the risk of HTN in an inverted V-shaped dose-response relationship (*p* for overall association = 0.023, non-linearity *p* = 0.009). Co showed no dose-response relationship with HTN (*p* for overall association = 0.106, non-linearity *p* = 0.299).

### 3.4. Combined effects of metal mixture

To further explore whether multiple metals had combined effects on the risk of HTN, we included all 13 metals in the BKMR model. Results showed that the overall effect of metal mixture was not statistically significant at or above the 25th percentile, compared to their median level (Supplementary Figure S6A).

The univariate-effect model of metals was analyzed by estimating univariate summaries of changes in HTN risk associated with changes in individual metals from the 25th percentile to the 75th percentile, while all other metals were held at a specific percentile (25th, 50th, or 75th percentile). We found that the effects of all single metals (changing from the 25th to the 75th percentile) on HTN risk were not statistically significant when all other metals were fixed at the 25th, 50th, or 75th percentile (Supplementary Figure S6B).

### 3.5. Subgroup analysis

Results of the subgroup analysis were shown in Supplementary Table S3. Compared to the first quartile, ORs for the third and fourth quartiles of Co levels were 0.60 (0.39, 0.91) and 0.63 (0.40, 0.97) in the females (*p*-trend < 0.05), and the interaction effect of gender was suggested for Co quartiles (*p*-trend < 0.05). The ORs for Co in the second quartile were 0.64 (0.42, 0.97), 0.69 (0.50, 0.96), and 0.69 (0.49, 0.96) for subjects aged more than 60 years, non-smokers and non-drinkers, respectively.

Compared to the lowest quartile of Al, the ORs in the second quartile were 0.62 (95% CI: 0.38, 0.99) in younger participants, while the OR in the third quartile was 0.58 (95% CI: 0.38, 0.90) in the population older than 60 years. The ORs for Al from the second to the third quartile were 0.48 (0.30, 0.77) and 0.61 (0.39, 0.93) in the low BMI group, respectively; whereas in the high BMI group, a significant association was only observed in the third quartile (OR 0.59, 95% CI: 0.36, 0.96). And there were negative associations between Al and risk of HTN in the females, non-smoker and non-drinker groups from the second to third quartiles. In addition, the OR for the third percentile was 1.69 (95% CI: 1.11, 2.57) for subjects aged over 60 years compared to the lowest quartile of Ca; and a significant association was only observed in the second percentile in the low BMI group (OR 1.64, 95% CI: 1.08, 2.50). No interaction effects were shown between either the quartiles of Al or Ca and the stratified factors.

## 4. Discussion

In this study, we applied plasma metals as the internal exposure indicator to explore the relationship between polymetals and HTN. Compared to other biological samples, plasma may be a reliable and stable long-term biomarker for the combined accumulation of metals in the human body (21). We found that the plasma levels of Co (second quartile: 0.21–0.25 μg/L) and Al (second and third quartiles: 18.10–36.70 μg/L) were negatively associated with the risk of HTN, and Ca (second quartile: 75,482.88–80,341.21 μg/L) was positively associated with the risk of HTN by the logistic regression analysis. The RCS model showed a non-linear dose-response relationship between plasma Al (V-shaped curve), Ca (inverted V-shaped curve) levels and the risk of HTN, respectively. BKMR analysis showed no joint effect of the metal mixture.

### 4.1. Cobalt

As a component of vitamin B12, cobalt is one of the essential trace elements indispensable for normal physiological function (22). Human exposure to Co includes environmental, dietary, occupational, and medical sources, whereas dietary intake (food and beverage) is a major source for the general population (22). A recent biokinetic modeling study on Co showed that Co concentration (300 μg/L or less) in the blood do not cause any adverse effects to humans, while exceeding 700 μg/L may lead to a more serious risk of reproductive, neurological or cardiac effects (23). Previous studies have shown that plasma Co concentration accurately reflect the level of Co exposure in humans and is a reliable biomarker of Co exposure (24). Compared to urine Co (half-life: 5.3 days), plasma Co (half-life: 9.1 days) is better in assessing Co levels due to its longer half-life and more stable property (25). Our study found that the plasma Co concentration in the non-hypertensive group was 0.25 μg/L, and a report of the elderly population in East China (Anhui Province) showed that the blood Co concentration in the general population was lower (0.14 μg/L) (26). A cross-sectional study from France reported that the blood Co concentration in French adults was 0.29 μg/L, which was slightly higher than the concentration reported in this study (27).

We found that Co had a protective effect on HTN in this study. In the single-metal model, the second quartile of plasma Co (0.21–0.25 μg/L, OR = 0.71) was inversely associated with the risk of HTN. Current epidemiological reports on the relationship between Co levels and risk of HTN still remain limited and contradictory. Consistent with our results, a prospective cohort study of 3,260 Chinese women (28) showed a linear and inverse correlation between blood Co levels and gestational HTN (mean values of 0.18 and 0.38 μg/L in the first and second trimester, respectively). Additionally, Wang et al. found that the Co content of hair was negatively associated with the risk of HTN for rural women from northern China (29). In contrast, a cross-sectional study of occupationally exposed workers in China found a significant increase in the prevalence of HTN when urine Co concentrations were above 2.3 μg/L (30). Meanwhile, a cohort study of 47,595 US women (31) found that people living in areas with higher Co exposure might have higher risk of HTN (median = 9.71 × 10^−6^ μg/m^3^, prevalence ratio = 1.03, 95% CI = 1.00, 1.07). Co, also known as a transition metal, is harmful to humans in deficiency or in excess (5). It has been shown that the dose-response curve between transition metals and the risk of cardiovascular events tends to have a *U*- or *J*-shaped distribution (5). Therefore, different concentrations of Co exposure could lead to inconsistency between our findings and those described above. Some animal and *in vitro* studies also explained the protective effect of Co on HTN. Moreover, Co can also stimulate the activity of vascular tension converting enzyme (ACE) and lead to decrease in blood pressure (32).

In subgroup analyses (Supplementary Table S3), we observed that the negative correlation between Co and HTN was more obvious in female participants. And there was a significant interaction between Co and gender. We consider that differences in the distribution of plasma Co concentrations between male (0.26 μg/L) and female (0.23 μg/L) populations probably account for this phenomenon. Previous studies have also reported that trace element levels are generally lower in females than in males in the general population (23). However, it is not clear how gender differences affect the protective effect of Co on HTN and further studies are needed to elucidate the mechanisms involved.

### 4.2. Aluminum

Aluminum has been widely used in consumer products (33). The general population is exposed to Al through food additives, drugs, and commodity packages (33). When Al enters the body, it is mainly excreted in the urine, while a small proportion can be gradually deposited in the blood with a half-life of several years, indicating that blood Al is a biological indicator of longer-term exposure (34). Previous experimental animal studies have generally concluded that Al may induce an increase in blood pressure (35, 36). Martinez et al. (36) found that Al exposure mainly promoted the increase of ROS of NAD (P) H oxidase and vascular prostaglandin COX-2, which induced vascular dysfunction through a synergistic effect and increased the blood pressure of rats. Zhang et al. (35) proposed that Al may induce HTN by interfering with the function of the erythrocyte membrane.

In the epidemiological studies, associations between Al and the development of cardiovascular diseases have been extensively studied (37). In an Australian prospective study, the miners who were exposed to Al dust by inhalation had higher cardiovascular mortality than the general population (38). Moreover, Weng et al. observed a positive correlation between blood Al levels and arterial stiffness among patients undergoing hemodialysis (39). In our study, a negative and V-shaped dose-response association was found between Al exposure levels and HTN risk in the middle-aged and older adults. In the single logistic regression model, ORs for plasma Al were 0.66 and 0.60 in the second quartile (18.10–24.59 μg/L) and third quartile (24.59–36.70 μg/L), respectively. This association remains consistent in the multi-metal logistic regression model. Notably, the association disappeared when the Al concentration was at a higher level (>36.70 μg/L). Nevertheless, epidemiological studies on Al exposure and the risk of HTN are quite limited. A study from Anhui Province, China, assessed the association between blood Al exposure and HTN in 1,013 elderly participants. However, they did not observe a correlation between blood Al (median: 62.24 μg/L) concentration levels and the risk of HTN (40). Furthermore,a study from the US cohort of 1,131 mother-infant pairs found no association between prenatal maternal exposure to environmental particulate Al (median: 56.63–57.61 ng/m^3^) and neonatal HTN (41). Additionally, in a recent cross-sectional study of Chinese elderly people (*n* = 1,013) (26), combined exposure to Al, Co, and vanadium was found to be a protective factor for dyslipidemia. Accordingly, Al could reduce the risk of HTN by modulating dyslipidemia (42). To our knowledge, our study is the first to identify a negative association between low plasma level of Al and the prevalence of HTN. We supposed that this negative correlation could be related to the compensatory mechanism stimulated by Al in the organism. One study found that lower concentrations of Al reduced vascular reactivity and promoted a compensatory increase in nitric oxide to dilate blood vessels (43). However, no experimental studies have yet focused on the differences in the effects of Al exposure on blood pressure at low or high metal concentrations, and further researches are needed to explore the mechanisms in the future.

### 4.3. Calcium

Calcium, an essential element, is an extremely vital component of the human body (44). Dietary Ca is the major source of among the general population (6). Experimental studies on the hemodynamics of HTN have found a direct link between total plasma Ca concentration and central or peripheral blood pressure (45). Plasma Ca is a good exposure biomarker in humans (45). The equilibrium of plasma Ca plays an important role in maintaining the physiological activities of the body, including the regulation of hormone secretion, nerve conduction, bone support and vascular activity, and is often used as an accurate indicator of Ca status in health and disease (44). The intake of Ca varies widely among different populations. In the United States, the recommended daily intake for adults (>18 years) is 1,000 and 1,200 mg/day of Ca is recommended for the elderly (>70 years) (6).

Currently the reports on the association between calcium exposure and HTN remain inconsistent. In a cross-sectional data from NHANES involving 26,778 US adults (46), higher concentrations of serum Ca (median: 94 mg/L) were found to be positively associated with HTN. In a prospective cohort study from China (47), Ca in the high quartile (means: 92–96.84 mg/L) was found to be significantly associated with an increased risk of HTN. High concentration of serum Ca can flow into arterial smooth muscle and directly promote vasoconstriction, increase vascular resistance and blood pressure (48). Nevertheless, Wang et al. (49) found a positive correlation between lower concentrations of Ca in plasma (median: 77.25 mg/L) and the risk of HTN for people aged 40–75 years in southwest China. Similarly, in our study, lower concentrations of plasma Ca (75.48–80.34 mg/L) increased the risk of HTN (OR: 1.40, 95% CI: 1.04–1.89). The mechanism may be related to Ca dysregulation, with negative feedback mechanisms at low blood Ca levels regulating the secretion of PTH in the body and stimulating the adrenal glands to secrete aldosterone (50), which would increase blood pressure. In addition, low blood Ca concentrations would lead to activation of the RAAS system, which would increase intracellular Ca concentrations and contribute to vasoconstriction (51). Additionally, our study observed an inverse V-shaped dose-response relationship between plasma Ca levels and the risk of HTN (*p* for overall association = 0.023, non-linear *p* = 0.009). Similarly, the 1991–2011 cohort study from the China Health and Nutrition Survey examined 1,611 participants and found a non-linear association between dietary calcium intake during adolescence and HTN in adulthood (*p* for overall association = 0.050, non-linearity *p* = 0.046) (52). However, relevant experimental studies are still lacking and the risk associations and mechanisms between different concentrations of plasma Ca and HTN need to be further explored. In fact, the middle-aged and elderly are often characterized by reduced Ca absorption (6), and it is recommended that dietary Ca supplementation should be taken into account for the elderly.

### 4.4. Metal mixtures

In our study, we used the BKMR statistical model to assess whether metal mixtures have a mixed effect on HTN risk. Although we did not find significant correlations, BKMR has advantages over traditional statistical models (logistic or linear regression) for analyzing multi-pollutant exposures. Epidemiological studies exploring the overall effect of metal mixtures on HTN using multi-pollution statistical methods are still limited. A study from long-term follow-up data from the US Gulf Coast (53) explored the association between blood metal mixtures (cadmium, mercury, manganese, lead, selenium) and HTN using a quantile-based g calculation. The results showed that there was no significant association between mixtures of blood cadmium, mercury, manganese, lead and selenium, and HTN (OR: 0.96, 95% CI: 0.73–1.27). In a Korean cross-sectional analysis of NHANES adults (54), the combined effect of three metals (Cd, Hg, and Pb) in whole blood on the risk of HTN was assessed using a weighted quantile sum (WQS) regression model. This showed that the WQS index with Pb as the major component was significantly and positively associated with HTN. In addition, Zhong et al. (9) investigated the association between urinary polymetallic mixtures and the risk of HTN in adults along the Yangtze River in China. Bayesian Kernel Machine Regression (BKMR) analysis revealed a significant association effect of five metals (Cd, Cu, Mg, Mo, and Zn) on HTN. However, data on plasma metals and risk of HTN is missing from other regions of China. Therefore, our cross-sectional study explored the single and combined effects of 13 plasma metals and HTN using the BKMR model. Although we did not find the combined effect of metal mixture on HTN risk, further longitudinal studies are needed to validate our findings.

### 4.5. Advantages and limitations

This study had a relatively large population and tested 13 metals simultaneously to assess the effects of multiple metals on human health. We used multiple statistical models to complement and validate our findings, ensuring the reliability of our conclusions. Furthermore, we employed the BKMR model and it has greater applicability in exploring the complex effects of human co-exposure to a mixture of chemicals. In addition, several limitations should also be taken into account in the elaboration of our study. First, several confounding factors were not collected in this study such as diet, exercise and medicines, although these unknown confounding effects have been assessed by applying E-values. Second, we cannot rule out reverse causal inferences resulting from cross-sectional studies, which would require further exploration in prospective studies. Furthermore, measuring plasma metal concentrations was probably not the most appropriate approach for some metals, since different metals may have different half-lives and characteristics, which may lead to misclassification and estimation bias. Blood samples may be suitable for measuring concentrations of metals such as calcium (45), while urine (cadmium), hair (mercury), and toenail (lead) have also been reported to be suitable for the measurement of some metals (21). Finally, the representation of the general population may be limited by only including adults over 30 years old in this study.

## 5. Conclusions

In this study, we found that low plasma cobalt and Aluminum were associated with the decreased risk of HTN among adults in Shenzhen, China, while low plasma calcium levels were associated with increased risk. Aluminum and calcium showed a V-shaped or an inverse V-shaped dose-response relationship with HTN, respectively. Moreover, we did not find the overall association between the 13 plasma metal mixtures and the risk of HTN. Our research may provide scientific evidence for the precision prevention of HTN, including formulation of environmental hygiene standards and the dietary guidance. Further studies are needed to confirm our findings and their potential mechanisms with prospective studies and experimental study designs.

## Data availability statement

The datasets presented in this article are not readily available to protect the privacy of study participants. Requests to access the datasets should be directed to SH, huangsuli420@163.com.

## Ethics statement

The studies involving human participants were reviewed and approved by Ethics Committee of the Shenzhen Center for Disease Control and Prevention. The patients/participants provided their written informed consent to participate in this study.

## Author contributions

SZ: data curation, formal analysis, methodology, writing—original draft, and writing—review and editing. ZN: methodology, validation, visualization, and writing—review and editing. ZLv: project administration, methodology, and writing—review and editing. TW: visualization, software, and writing—original draft. WW: formal analysis and conceptualization. DF, XZ, YF, and TC: methodology and validation. ZLi, QL, and HH: data curation and visualization. YW: investigation, data curation, project administration, writing—original draft, and resources. SH: data curation, supervision, writing—review and editing, methodology, and funding acquisition. All authors contributed to the article and approved the submitted version.
