# Supplementary material for: Associations between plasma metal mixture exposure and risk of hypertension: A cross-sectional study among adults in Shenzhen, China
Source: Front Public Health. 2022 Dec 13;10:1039514. doi: 10.3389/fpubh.2022.1039514 (PMC9794142; doi:10.3389/fpubh.2022.1039514)
Supplement: Supplementary file 1 [file Data_Sheet_1.docx]

**Supporting Information**

**Associations between plasma metal mixture exposure and risk of hypertension: a cross-sectional study among adults in Shenzhen, China**

Sijia Zheng^1, 2^, †, Zhiqiang Nie^3^, †, Ziquan Lv^2^, Tian Wang^4, 2^, Weizhou Wei^5^, Daokui Fang^2^, Xuan Zou^2^, Yulin Fu^2^, Tingting Cao^2^, Zhaoyi Liang^1, 2^, Qi Lu^1, 2^, Hui Huang^6^, Ying Wen^2, **^, Suli Huang^1, 2, *^

^1^ School of Public Health, Shanxi Medical University, Taiyuan 030001, China

^2^ Shenzhen Center for Disease Control and Prevention, Shenzhen, 518055, China

^3^ Department of Cardiology, Guangdong Cardiovascular Institute, Guangdong Provincial People’s Hospital, Guangdong Academy of Medical Sciences, Guangzhou, 510010, China

^4^ Key Laboratory of Environmental Medicine Engineering, Ministry of Education, School of Public Health, Southeast University, Nanjing 210009, China

^5^ Shenzhen Yutian Community Health Service Centre, Shenzhen, 518036, China

^6^ Department of Cardiology, The Eighth Affiliated Hospital, Sun Yat-sen University, Shenzhen, 518033, China

Correspondence to:

S. Huang, School of Public Health, Shanxi Medical University; Shenzhen Center for Disease Control and Prevention, Shenzhen, 518055, China.

Y. Wen, Shenzhen Center for Disease Control and Prevention, Shenzhen, 518055, China.

^†^ The authors contributed equally to this study.

^*, **^Corresponding author

**Appendix S1: *Information collection***(Wen et al., 2019)

We defined diabetes as fasting glucose≥7.0 mmol/L, or self-reported history of diabetes diagnosis, or use of antidiabetic drug. Hyperlipidemia was defined when one of the following criteria was met: total cholesterol≥6.2 mmol/L, triglycerides≥2.3 mmol/L, low density lipoprotein cholesterol (LDLC)≥4.1 mmol/L, self-reported antihyperlipidemia medication use, or physician's diagnosis. Smokers were those who smoked daily and smoked for over half a year, including current or former smokers. Participants were classified as alcohol drinkers if they reported drinking alcohol at least once a week and had an alcohol drinking history of over half a year.

**Appendix S2: *Plasma metal concentration detection***(Wen et al., 2019)

The plasma metals were measured using the inductively coupled plasma mass spectrometry (ICP-MS, Agilent 7700 series, Agilent Technologies, USA) as previously reported (Wen et al., 2019).

We used the wet acid digestion method to digest blood samples. Firstly, plasma sample was incubated with 300 μL 55% (v/v) HNO3 (TAMAPURE-AA 10 ultrapure analytical reagent, Tamachemicals CO., Kawasaki, Japan) at room temperature for 2 h, then the samples were transferred to the water bath at boiling temperature until the solution turned light yellow. After cooling, the sample was diluted to 6.0 mL with ultrapure water for final analysis. The samples were analyzed in a random order, and the sample information was blind to the laboratory staff. We used the certified reference materials (ClinChek human plasma controls for trace elements no. 8883 and no. 8884, Recipe, Munich, Germany) to evaluate the accuracy and precision of the detection. The measured values of the reference materials were within the recommended ranges for each metal. Moreover, standard reference materials 1640a (Trace Elements in Natural Water, National Institute of Standards and Technology, Gaithersburg, MD, USA) were analyzed to assess the instrument performance every 30 samples.If the measured values differed from the certifified values or indicated potential contamination, the instrument was recalibrated and the previous samples were reanalyzed.

**Reference**

Wen, Y., Huang, S., Zhang, Y., Zhang, H., Zhou, L., Li, D., Xie, C., Lv, Z., Guo, Y., Ke, Y., Wu, T., Cheng, J. 2019. Associations of multiple plasma metals with the risk of ischemic stroke: A case-control study. Environ Int. 125, 125-134. doi:10.1016/j.envint.2018.12.037

**Table of Contents**

**Table S1.** Limits of detection, distributions of plasma metals among the study population, intra-assay and inter-assay coefficients of variation (n=1546)

**TableS2** Multiple linear regression between SBP, DBP and plasma metals

**Table S3.** Stratification analyses for associations between plasma metals and hypertension.

**Figure S1.**Flow chart of the inclusion of study participants.

**Figure S2.** Directed acyclic graph (DAG) diagrams from the Metals and Hypertension

Association Study.

**Figure S3.** Correlations between plasma metals among the study population (n=1546)

**Figure S4.** E-values for the ORs of Hypertension in the single-metal models.

**Figure S5.** The multiple-metal model for the associations of plasma metal quartiles with the risk of hypertension.

**Figure S6.** Associations between 13 metals and hypertension by BKMR model.

| **Table S1. Limits of detection, distributions of plasma metals among the study population, intra-assay and inter-assay coefficients of variation (n=1546)** | | | | | | | | | | | |
| --- | --- | --- | --- | --- | --- | --- | --- | --- | --- | --- | --- |
| **Plasma metals (μg/L)** | **LOD** | **Total No. (%) <LOD** a | **non-HTN <LOD** a | **HTN <LOD** a | **5th percentile** | **25th percentile** | **50th percentile** | **75th percentile** | **95th percentile** | **Intra-assay CV%** | **Inter-assay CV%** |
| Magnesium | 100 | 0 (0.00) | 0 (0.00) | 0 (0.00) | 16579.37 | 18823.48 | 20570.65 | 22325.68 | 24878.05 | 0.05 | 0.08 |
| Manganese | 0.2 | 67 (4.33) | 36(3.98) | 31 (4.83) | 0.22 | 0.6 | 0.94 | 1.44 | 3.07 | 7.20 | 8.97 |
| Cobalt | 0.01 | 1 (0.07) | 1 (0.11) | 0 (0.00) | 0.09 | 0.2 | 0.24 | 0.29 | 0.39 | 4.39 | 5.87 |
| Aluminum | 1.00 | 8(0.53) | 3 (0.34) | 5(0.80) | 9.80 | 16.97 | 23.90 | 37.29 | 69.68 | 4.75 | 6.94 |
| Iron | 100.00 | 0 (0.00) | 0 (0.00) | 0 (0.00) | 904.95 | 1313.75 | 1693.43 | 2204.25 | 3334.52 | 5.92 | 7.47 |
| Calcium | 1000.00 | 0 (0.00) | 0 (0.00) | 0 (0.00) | 67586.07 | 75143.44 | 79798.37 | 86986.65 | 102717.74 | 0.06 | 0.08 |
| Copper | 5.00 | 0 (0.00) | 0 (0.00) | 0 (0.00) | 615.26 | 777.76 | 919.65 | 1052.70 | 1296.57 | 3.76 | 4.39 |
| Zinc | 300.00 | 0 (0.00) | 0 (0.00) | 0 (0.00) | 699.18 | 884.31 | 1049.01 | 1238.52 | 1555.45 | 3.77 | 4.08 |
| Arsenic | 0.03 | 0(0.00) | 0 (0.00) | 0 (0.00) | 0.33 | 0.64 | 1.14 | 2.08 | 4.70 | 7.55 | 8.71 |
| Selenium | 0.30 | 0 (0.00) | 0 (0.00) | 0 (0.00) | 71.27 | 87.14 | 98.93 | 113.66 | 139.60 | 3.48 | 4.54 |
| Molybdenum | 0.10 | 0 (0.00) | 0 (0.00) | 0 (0.00) | 0.63 | 0.87 | 1.07 | 1.34 | 1.90 | 1.41 | 6.48 |
| Cadmium | 0.01 | 96 (6.38) | 60(6.86) | 36(5.71) | 0.01 | 0.03 | 0.05 | 0.07 | 0.12 | 8.61 | 7.17 |
| Thallium | 0.01 | 0 (0.00) | 0 (0.00) | 0 (0.00) | 0.06 | 0.09 | 0.11 | 0.15 | 0.22 | 7.48 | 8.36 |

Note: LOD, limit of detection; CV, coefficient of variance.

Data are presented as number (percentage).

| **Table S2. Multiple linear regression between SBP, DBP and plasma metals** | | | | | |
| --- | --- | --- | --- | --- | --- |
| **Metal** | **SBP** | |  | **DBP** | |
|  | **β (95%CI)** | ***P*-value** |  | **β (95%CI)** | ***P*-value** |
| Mg | -1.663(-8.454,5.127) | 0.631 |  | -0.155(-4.506,4.196) | 0.944 |
| Mn | -0.080(-1.136,0.977) | 0.882 |  | 0.0126( -0.664,0.689) | 0.971 |
| Co | -1.219(-3.142, 0.704) | 0.214 |  | 0.434( -0.794,1.663) | 0.488 |
| Al | -1.565(-3.000,-0.129) | 0.033 |  | -1.294(-2.210,-0.378) | 0.006 |
| Fe | 1.014(-1.322,3.350) | 0.395 |  | 1.619(-0.125,3.113) | 0.134 |
| Ca | 0.074( -7.148,7.001) | 0.984 |  | 2.007( -2.524, 6.539) | 0.385 |
| Cu | 2.026(-1.982, 6.033) | 0.322 |  | 1.475(-1.089,4.039) | 0.259 |
| Zn | 0.868(-2.695, 4.431) | 0.633 |  | -0.757(-3.040,1.525) | 0.515 |
| As | 0.086(-0.995, 1.168) | 0.875 |  | 0.131( -0.565,0.827) | 0.712 |
| Se | 0.442(-3.748, 4.631) | 0.836 |  | 2.210(-0.474,4.894) | 0.107 |
| Mo | -0.047(-2.716, 2.621) | 0.972 |  | -0.795( -2.507,0.916) | 0.362 |
| Cd | 0.520(-1.043, 2.082) | 0.514 |  | 0.278( -0.721, 1.276) | 0.586 |
| Tl | 1.158(-0.976,3.292) | 0.287 |  | -0.305(-1.674,1.065) | 0.662 |
| Note:Adjusted factors included age, sex, BMI, smoking, and drinking, UA, family history of hypertension, diabetes, hyperlipidemia and eGFR. Ln-transformed plasma metal concentrations were used as continuous variables. | | | | | |
|  |  |  |  |  |  |

| **TableS3. Odds ratios (95% coefficient intervals) for hypertension in subgroups stratified by gender, age, body mass index ,smoking and drinking** | | | | | | |
| --- | --- | --- | --- | --- | --- | --- |
|  |  |  |  |  |  |  |
| **Variables** | **Q1** | **Q2** | **Q3** | **Q4** | ***p*-trend ^a^** | ***p*-interaction ^b^** |
| **Cobalt** |  |  |  |  |  |  |
| Male(n=701) |  |  |  |  |  |  |
| n (controls/cases) | 103/82 | 104/61 | 103/66 | 103/72 |  |  |
| OR(95%CI) | 1.00 (reference) | 1.04 (0.64, 1.69) | 1.08 (0.67, 1.73) | 1.34 (0.83, 2.16) | 0.260 | 0.014 |
| Female(n=845) |  |  |  |  |  |  |
| n (controls/cases) | 114/129 | 114/91 | 114/71 | 114/63 |  |  |
| OR(95%CI) | 1.00 (reference) | 0.73 (0.49, 1.10) | 0.60 (0.39, 0.91) | 0.63 (0.40, 0.97) | 0.011 |  |
| Age<60(n=769) |  |  |  |  |  |  |
| n (controls/cases) | 128/75 | 128/53 | 128/56 | 128/39 |  |  |
| OR(95%CI) | 1.00 (reference) | 0.99 (0.61, 1.60) | 1.04 (0.65, 1.66) | 0.90 (0.54, 1.51) | 0.785 | 0.977 |
| Age≥60(n=776) |  |  |  |  |  |  |
| n (controls/cases) | 89/129 | 90/86 | 89/92 | 89/105 |  |  |
| OR(95%CI) | 1.00 (reference) | 0.64 (0.42, 0.97) | 0.75 (0.49, 1.14) | 0.83 (0.55, 1.26) | 0.306 |  |
| BMI<24(n=830) |  |  |  |  |  |  |
| n (controls/cases) | 127/95 | 127/65 | 127/76 | 126/60 |  |  |
| OR(95%CI) | 1.00 (reference) | 0.80 (0.51, 1.24) | 1.09 (0.70, 1.67) | 1.00 (0.64, 1.58) | 0.839 | 0.893 |
| BMI≥24(n=716) |  |  |  |  |  |  |
| n (controls/cases) | 90/123 | 91/74 | 91/66 | 90/76 |  |  |
| OR (95% CI) | 1.00 (reference) | 0.75 (0.47, 1.17) | 0.66 (0.42, 1.04) | 0.79 (0.50, 1.24) | 0.166 |  |
| Smoking (n=194) |  |  |  |  |  |  |
| n (controls/cases) | 33/14 | Oct-34 | 34/15 | 33/19 |  |  |
| OR (95% CI) | 1.00 (reference) | 0.73 (0.24, 2.28) | 1.03 (0.35, 2.97) | 1.98 (0.69, 5.73) | 0.173 | 0.098 |
| Non-smoking (n=1333) |  |  |  |  |  |  |
| n (controls/cases) | 181/197 | 181/126 | 181/131 | 180/116 |  |  |
| OR (95% CI) | 1.00 (reference) | 0.69 (0.50, 0.96) | 0.79 (0.57, 1.10) | 0.74 (0.53, 1.04) | 0.068 |  |
| Drinking (n=180) |  |  |  |  |  |  |
| n (controls/cases) | 26/25 | 25/19 | 27/10 | 25/20 |  |  |
| OR (95% CI) | 1.00 (reference) | 1.14 (0.45, 2.90) | 0.66 (0.23, 1.85) | 0.92 (0.35, 2.38) | 0.672 | 0.94 |
| Non-drinking (n=1343) |  |  |  |  |  |  |
| n (controls/cases) | 188/187 | 188/119 | 188/126 | 188/120 |  |  |
| OR (95% CI) | 1.00 (reference) | 0.69 (0.49, 0.96) | 0.80 (0.58, 1.11) | 0.84 (0.60, 1.17) | 0.246 |  |
| **Variables** | **Q1** | **Q2** | **Q3** | **Q4** | ***p*-trend ^a^** | ***p*-interaction ^b^** |
| **Aluminum** |  |  |  |  |  |  |
| Male(n=701) |  |  |  |  |  |  |
| n (controls/cases) | 102/91 | 103/61 | 103/49 | 102/69 |  |  |
| OR(95%CI) | 1.00 (reference) | 0.91 (0.57, 1.47) | 0.80 (0.49, 1.33) | 0.93 (0.58, 1.48) | 0.683 | 0.627 |
| Female(n=845) |  |  |  |  |  |  |
| n (controls/cases) | 118/122 | 118/63 | 118/69 | 118/100 |  |  |
| OR(95%CI) | 1.00 (reference) | 0.52 (0.33, 0.79) | 0.51 (0.34, 0.78) | 0.76 (0.50, 1.13) | 0.241 |  |
| Age<60(n=769) |  |  |  |  |  |  |
| n (controls/cases) | 131/75 | 132/48 | 132/44 | 131/54 |  |  |
| OR(95%CI) | 1.00 (reference) | 0.62 (0.38, 0.99) | 0.74 (0.46, 1.20) | 0.80 (0.50, 1.28) | 0.461 | 0.446 |
| Age≥60(n=776) |  |  |  |  |  |  |
| n (controls/cases) | 89/118 | 89/93 | 89/77 | 88/115 |  |  |
| OR(95%CI) | 1.00 (reference) | 0.77 (0.50, 1.17) | 0.58 (0.38, 0.90) | 0.90 (0.59, 1.36) | 0.526 |  |
| BMI<24(n=830) |  |  |  |  |  |  |
| n (controls/cases) | 130/107 | 130/51 | 130/66 | 129/69 |  |  |
| OR(95%CI) | 1.00 (reference) | 0.48 (0.30, 0.77) | 0.61 (0.39, 0.93) | 0.69 (0.45, 1.06) | 0.128 | 0.306 |
| BMI≥24(n=716) |  |  |  |  |  |  |
| n (controls/cases) | 91/106 | 91/75 | 91/54 | 90/96 |  |  |
| OR (95% CI) | 1.00 (reference) | 0.82 (0.52, 1.29) | 0.59 (0.36, 0.96) | 0.99 (0.64, 1.54) | 0.794 |  |
| Smoking (n=194) |  |  |  |  |  |  |
| n (controls/cases) | 33/16 | Dec-34 | 34/13 | 33/15 |  |  |
| OR (95% CI) | 1.00 (reference) | 2.48 (0.77, 7.93) | 2.39 (0.78, 7.33) | 1.97 (0.66, 5.87) | 0.295 | 0.624 |
| Non-smoking (n=1333) |  |  |  |  |  |  |
| n (controls/cases) | 184/192 | 185/116 | 185/98 | 184/155 |  |  |
| OR (95% CI) | 1.00 (reference) | 0.61 (0.44, 0.86) | 0.53 (0.38, 0.74) | 0.81 (0.59, 1.11) | 0.170 |  |
| Drinking (n=180) |  |  |  |  |  |  |
| n (controls/cases) | 26/27 | 27/12 | 26/13 | 26/19 |  |  |
| OR (95% CI) | 1.00 (reference) | 0.61 (0.23, 1.61) | 0.45 (0.17, 1.19) | 0.61 (0.24, 1.56) | 0.196 | 0.482 |
| Non-drinking (n=1343) |  |  |  |  |  |  |
| n (controls/cases) | 191/182 | 192/110 | 191/107 | 191/145 |  |  |
| OR (95% CI) | 1.00 (reference) | 0.63 (0.45, 0.88) | 0.63 (0.45, 0.89) | 0.85 (0.61, 1.17) | 0.375 |  |
| **Variables** | **Q1** | **Q2** | **Q3** | **Q4** | ***p*-trend ^a^** | ***p*-interaction ^b^** |
| **Calcium** |  |  |  |  |  |  |
| Male(n=701) |  |  |  |  |  |  |
| n (controls/cases) | 105/81 | 105/88 | 105/72 | 105/40 |  |  |
| OR(95%CI) | 1.00 (reference) | 1.33 (0.84, 2.09) | 1.24 (0.77, 2.00) | 0.88 (0.52, 1.50) | 0.56 | 0.911 |
| Female(n=845) |  |  |  |  |  |  |
| n (controls/cases) | 121/103 | 121/103 | 121/85 | 121/70 |  |  |
| OR(95%CI) | 1.00 (reference) | 1.34 (0.90, 2.02) | 1.15 (0.76, 1.75) | 0.82 (0.52, 1.30) | 0.345 |  |
| Age<60(n=769) |  |  |  |  |  |  |
| n (controls/cases) | 135/74 | 136/70 | 135/50 | 135/34 |  |  |
| OR(95%CI) | 1.00 (reference) | 0.95 (0.61, 1.47) | 0.86 (0.53, 1.38) | 0.73 (0.43, 1.23) | 0.215 | 0.085 |
| Age≥60(n=776) |  |  |  |  |  |  |
| n (controls/cases) | 90/100 | 91/108 | 91/133 | 90/73 |  |  |
| OR(95%CI) | 1.00 (reference) | 1.32 (0.86, 2.02) | 1.69 (1.11, 2.57) | 0.85 (0.53, 1.36) | 0.652 |  |
| BMI<24(n=830) |  |  |  |  |  |  |
| n (controls/cases) | 132/81 | 133/99 | 133/75 | 132/45 |  |  |
| OR(95%CI) | 1.00 (reference) | 1.64 (1.08, 2.50) | 1.33 (0.86, 2.08) | 0.84 (0.51, 1.38) | 0.354 | 0.497 |
| BMI≥24(n=716) |  |  |  |  |  |  |
| n (controls/cases) | 93/101 | 94/93 | 94/96 | 93/52 |  |  |
| OR (95% CI) | 1.00 (reference) | 1.22 (0.78, 1.91) | 1.32 (0.84, 2.07) | 0.75 (0.46, 1.25) | 0.329 |  |
| Smoking (n=194) |  |  |  |  |  |  |
| n (controls/cases) | 34/17 | 34/17 | 34/16 | 34/8 |  |  |
| OR (95% CI) | 1.00 (reference) | 1.38 (0.48, 3.97) | 1.77 (0.63, 4.91) | 0.64 (0.19, 2.17) | 0.421 | 0.998 |
| Non-smoking (n=1333) |  |  |  |  |  |  |
| n (controls/cases) | 189/160 | 189/167 | 189/158 | 189/92 |  |  |
| OR (95% CI) | 1.00 (reference) | 1.37 (0.99, 1.89) | 1.32 (0.95, 1.84) | 0.79 (0.55, 1.14) | 0.2 |  |
| Drinking (n=180) |  |  |  |  |  |  |
| n (controls/cases) | 26/17 | 27/37 | 27/14 | 26/6 |  |  |
| OR (95% CI) | 1.00 (reference) | 2.49 (0.99, 6.30) | 1.17 (0.42, 3.28) | 0.61 (0.18, 2.14) | 0.162 | 0.186 |
| Non-drinking (n=1343) |  |  |  |  |  |  |
| n (controls/cases) | 196/161 | 196/158 | 196/144 | 196/96 |  |  |
| OR (95% CI) | 1.00 (reference) | 1.34 (0.97, 1.85) | 1.24 (0.89, 1.72) | 0.83 (0.58, 1.19) | 0.258 |  |
| p-interaction, p-Values for the interaction terms. Subgroup analysis was conducted using logistic regression. Model by including the metals and other potential risk factors consistent with the multiple-metal model (except for the corresponding stratification variable).  ^a^ p-Values for trend test were obtained from the logistic regression models by using the median of each metal quartile (ln-transformed plasma metal concentrations) as a continuous variable.  ^b^ The interaction was examined by adding an interaction term between a specific metal and the stratification variable as well as the metals and other risk factors which were consistent with the multiple-metal model. | | | | | | |
|  |  |  |  |  |  |  |
|  |  |  |  |  |  |  |
|  |  |  |  |  |  |  |
|  |  |  |  |  |  |  |
|  |  |  |  |  |  |  |
|  |  |  |  |  |  |  |
|  |  |  |  |  |  |  |


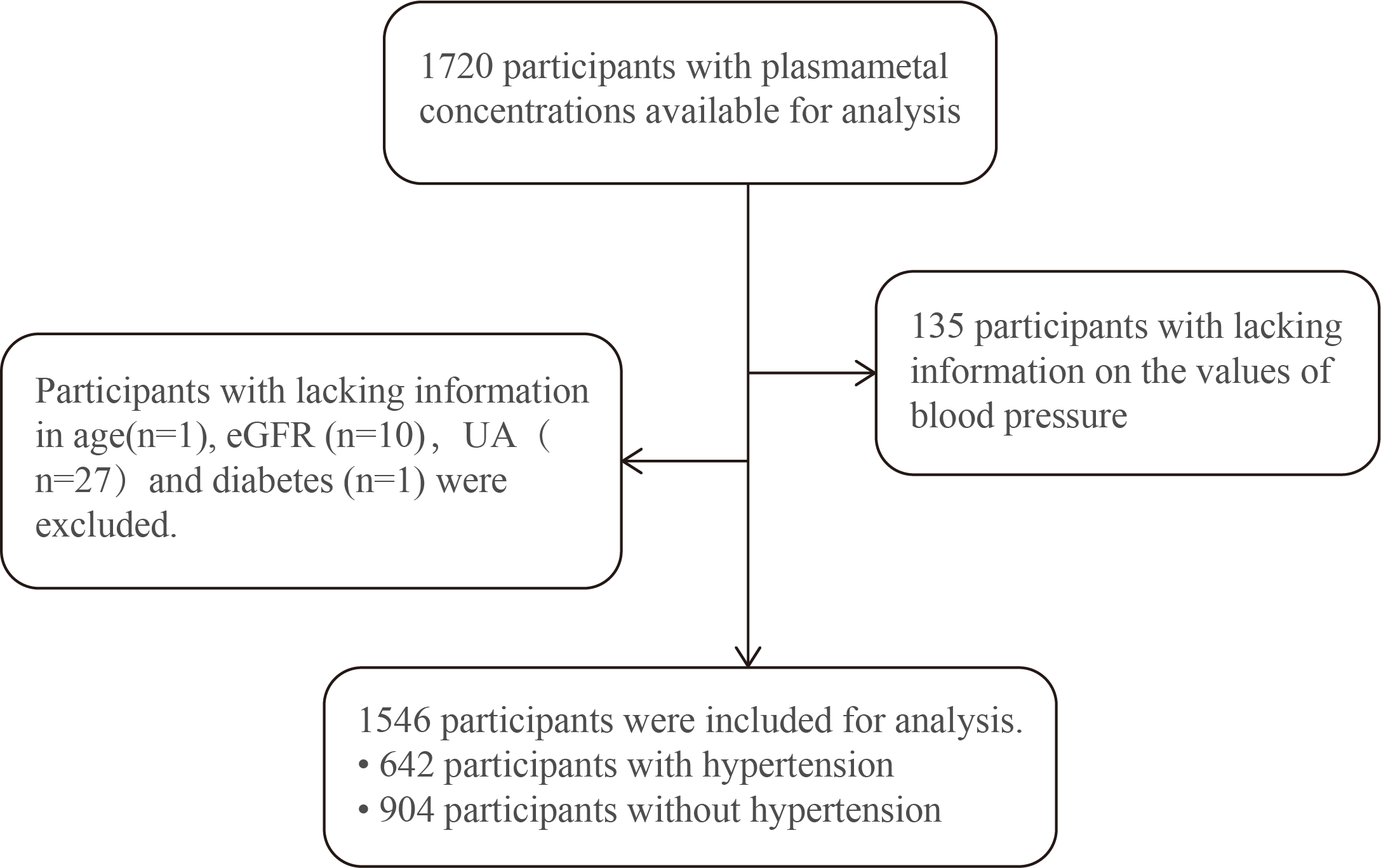


**Figure S1. Flow chart for inclusion of study participants (n=1546).**


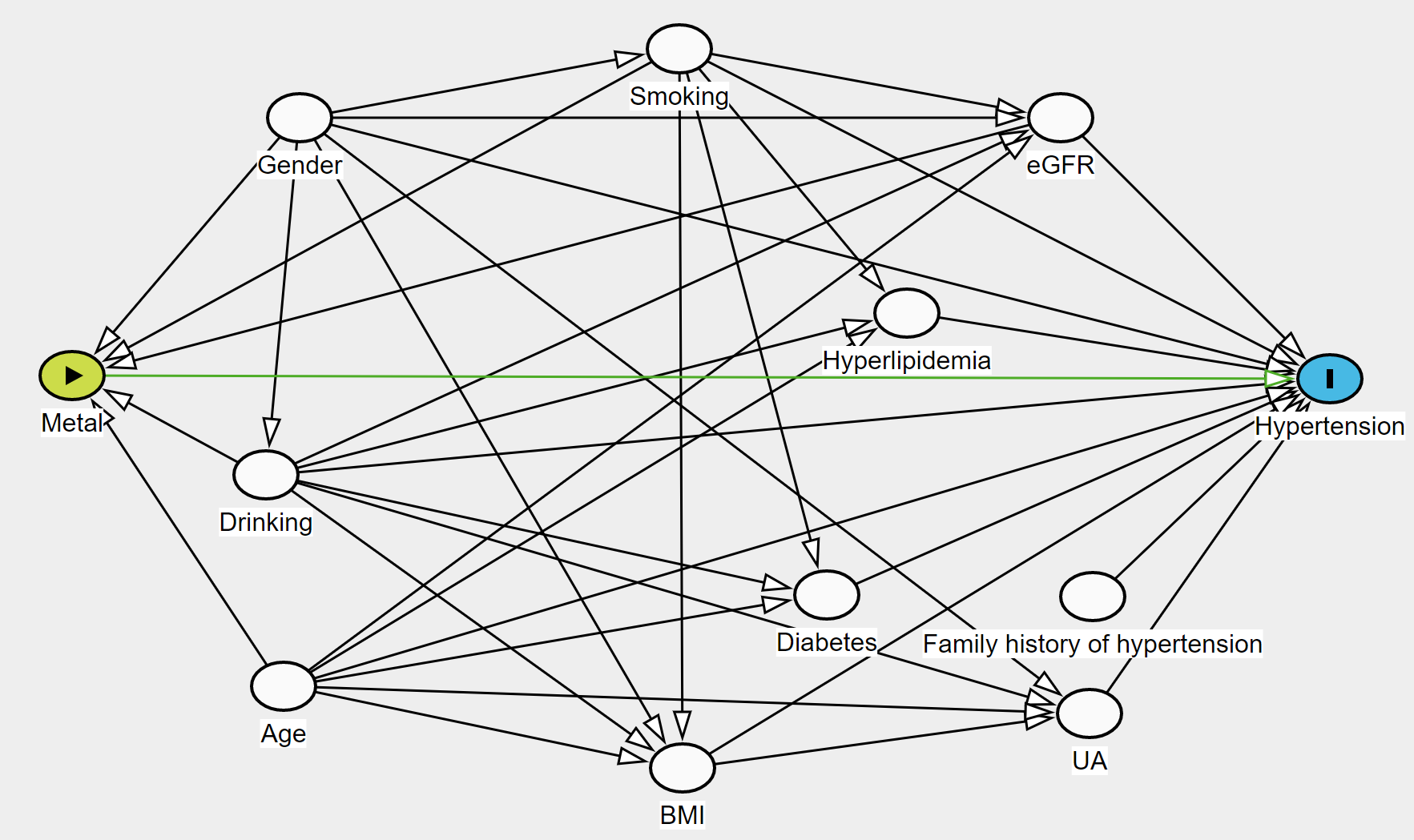


**Figure S2. Directed acyclic graph (DAG) diagrams from the Metals and Hypertension Association Study.** Nodes represent variables and arrows indicate causality. BMI, body mass index; UA, uric acide; eGFR, estimated glomerular filtration rate.


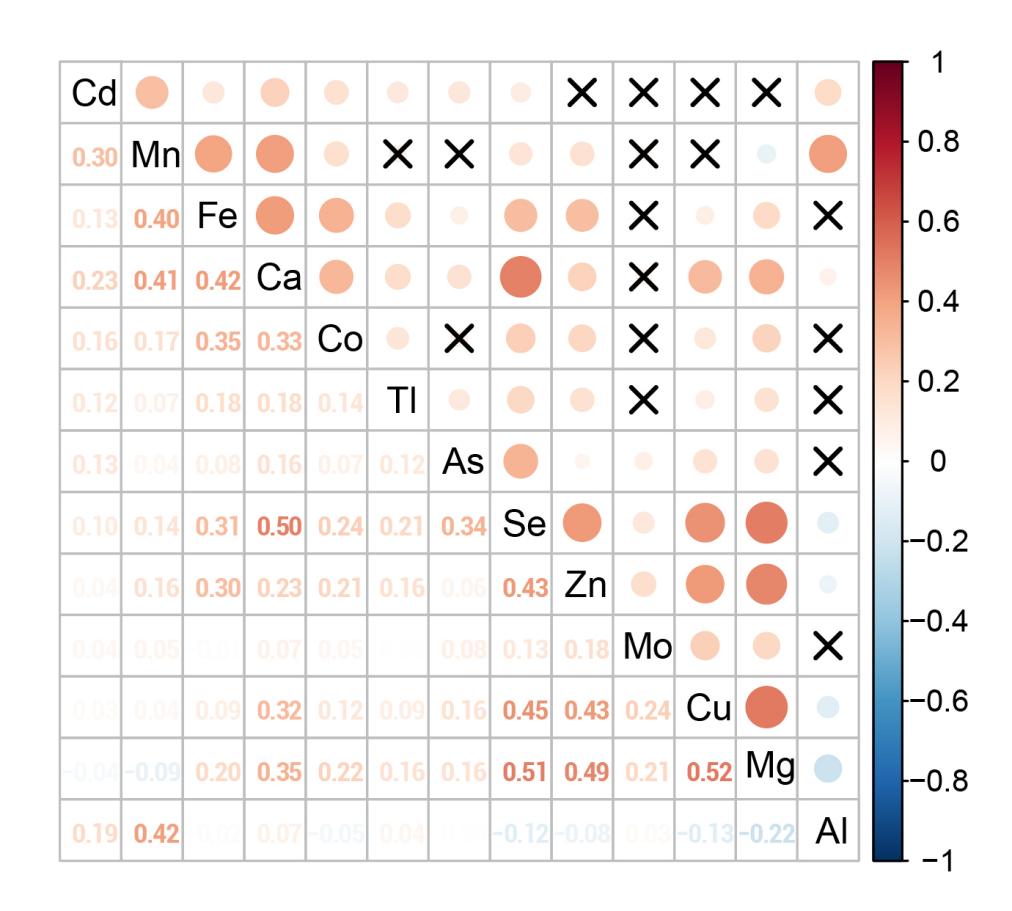


**Figure S3. Correlations between plasma metals among the study population (n=1546).** Al, Aluminum; As, Arsenic; Cd, Cadmium; Ca, Calcium; Co, Cobalt; Cu, Copper; Fe, Iron; Mg, Magnesium; Mn, Manganese; Mo, Molybdenum; Se, Selenium; Tl, Thallium; Zn, Zinc. Spearman’s rank correlation coefficients are presented. Plasma metals concentration were prior to transformed by the natural logarithm (ln). The colors and areas of circles reflect the values of corresponding correlation coefficients.

**
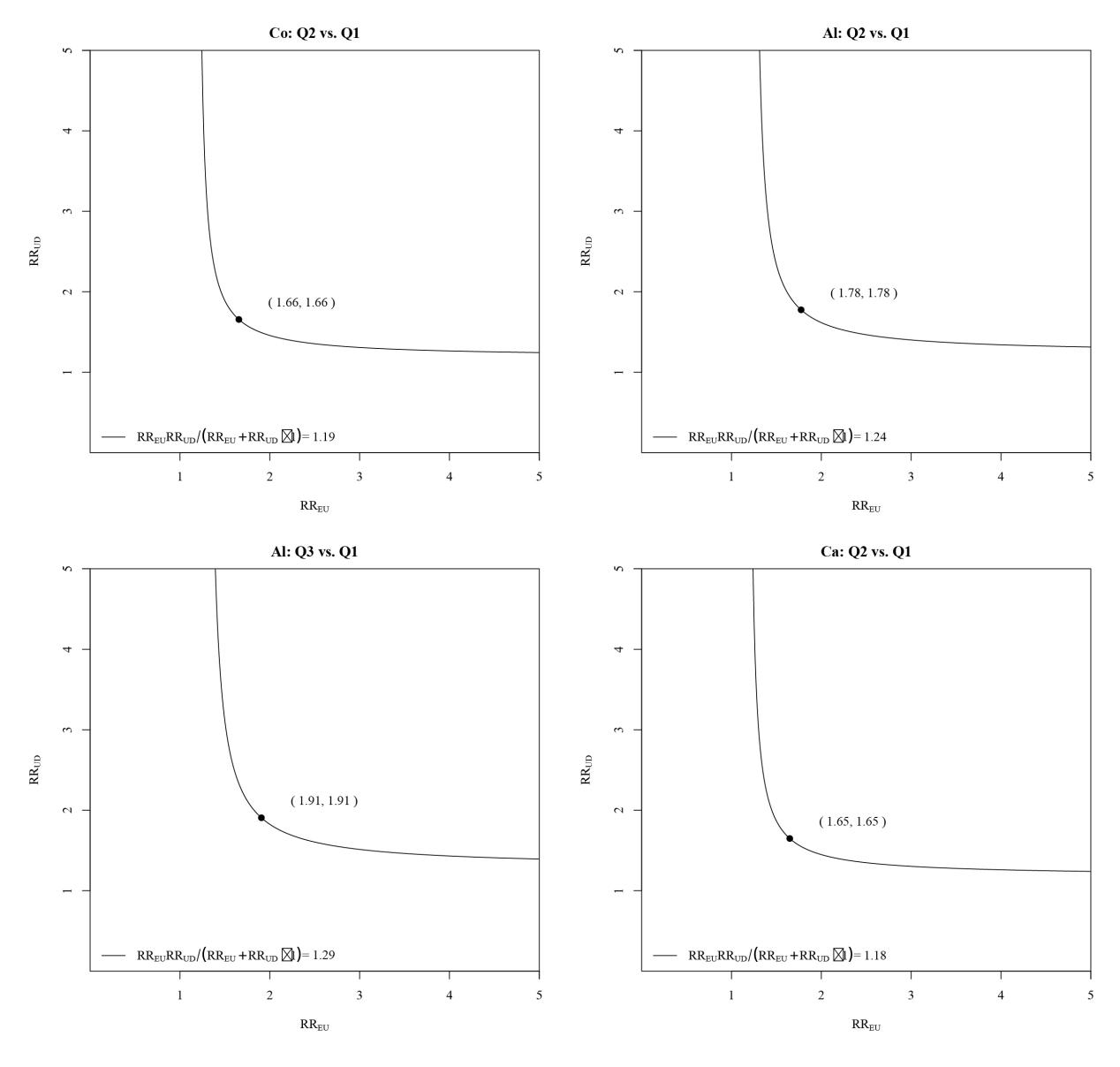
**

**Figure. S4 E-values for the ORs of Hypertension in the single-metal models.**

U, D and E represent unmeasured confounders, the outcome and exposure, respectively. RRUD is explained as the maximum risk ratio for the outcome comparing any 2 categories of the unmeasured confounders, with adjustment already made for the measured covariates; RREU is explained as the maximum risk ratio for any specific level of the unmeasured confounders comparing those with and without treatment, with adjustment already made for the measured covariates. Co, cobalt; Al, Aluminum Ca, Calcium. Q2 *vs*. Q1, the second quartile of plasma metal versus the lowest quartile;Q3 *vs.* Q1, the third quartiles of plasma metals versus the lowest quartiles.


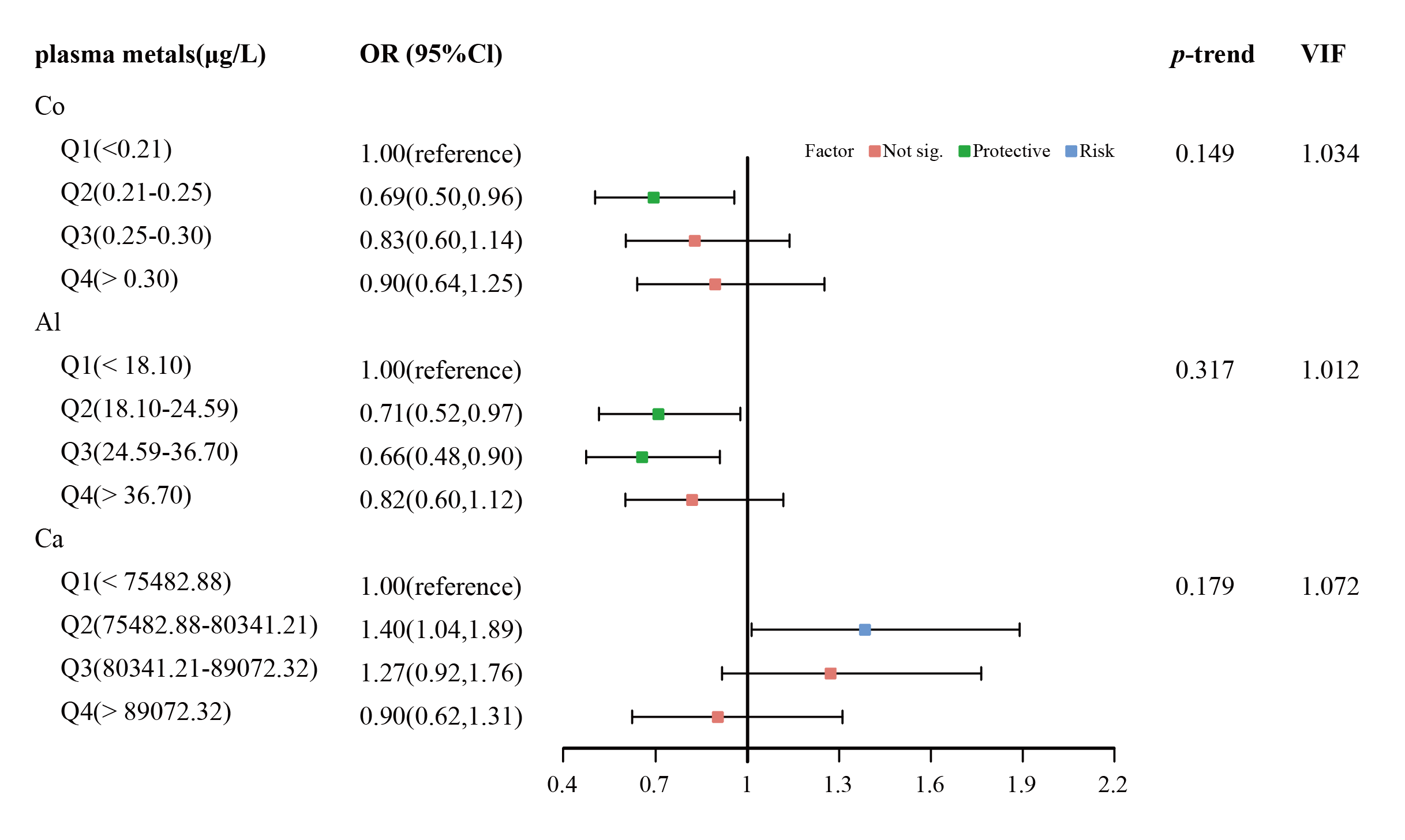


**Figure S5**. **The multiple-metal model for the associations of plasma metal quartiles with the risk of hypertension.**

OR, odds ratio; 95% CI, 95% confidence interval; *p*-trend, *p*-values for trend test; VIF, Variance Inflation Factor. In the multiple-metal model, metals significant in the single-metal model were analyzed by stepwise regression with adjustment for age, gender, BMI, smoking, alcohol drinking, UA, family history of hypertension, diabetes, hyperlipidemia and eGFR. The squares represent adjusted odds ratios, and the horizon lines represent 95% confidence intervals. *p*-Values for trend test were obtained from the logistic regression models by using the median of each metal quartile (ln-transformed plasma metal concentrations) as a continuous variable


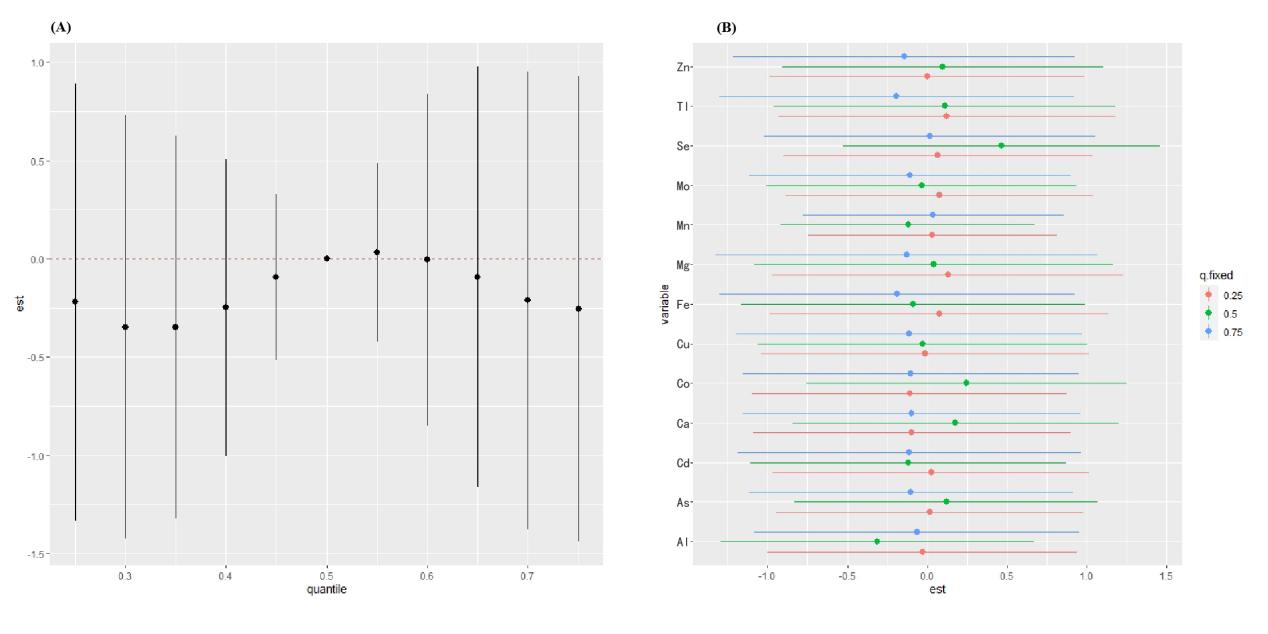


**Figure S6. Associations between 13 metals and hypertension by BKMR model.**

Data were estimated by Bayessian Kermel Machine Regression, while adjusting for factors were consistent with model 2 of the logistic regression. (A) Overall effect of the mixture estimates and 95% credible interval (B) Single metal association (estimate and 95% credible intervals). This plot estimates the risk of HTN for a single metal set at the 75th percentile versus the 25th percentile, when all other metals are fixed at the 25th percentile (red line), 50th percentile (green line) or 75th percentile (blue line).
